# Myocardial oxygenation is reduced in end-stage renal failure: a novel blood oxygen level dependant (BOLD)-cardiac MRI study

**DOI:** 10.1186/1532-429X-16-S1-P184

**Published:** 2014-01-16

**Authors:** Susie F Parnham, Suchi Grover, Craig Bradbrook, Darryl Leong, Carmine De Pasquale, Jonathan Gleadle, Joseph Selvanayagam

**Affiliations:** 1 Cardiology, Flinders University, Flinders Medical Centre, Bedford Park, South Australia, Australia; 2 Renal Medicine, Flinders Medical Centre, Bedford Park, South Australia, Australia; 3 Cardiovascular Magnetic Resonance Research, Flinders Medical Centre, Bedford Park, South Australia, Australia

## Background

Cardiovascular disease is the leading cause of mortality and morbidity in end-stage renal failure (ESRF) population, mostly from coronary artery disease (CAD). Majority of CAD in ESRF patients is asymptomatic and current cardiac stress imaging modalities are sub-optimal as risk predictors. Advances in cardiovascular magnetic resonance (CMR) with the novel blood oxygen level-dependent (BOLD) technique provides unprecedented capability to assess regional myocardial deoxygenation. We hypothesized that myocardial oxygenation would be reduced in ESRF patients and may form a novel strategy to assess myocardial ischemia.

## Methods

Sixteen chronic renal failure (CRF) patients (7 on dialysis, 9 pre-dialysis) with no known history of CAD underwent CMR scanning at 3.0 T. Given known reductions in BOLD signals in hypertrophied myocardium, we also assessed a control group of HT patients with no history of CAD (n = 6) Myocardial function, rest and stress BOLD was performed. To measure oxygenation, using a T2-prepared BOLD sequence, myocardial Signal Intensity (SI) was measured at adenosine stress (140 μg/kg/min) and at rest (corrected to RR interval). Comparison of myocardial SI analyses were performed using multivariate linear regression.

## Results

**Figure 1:**
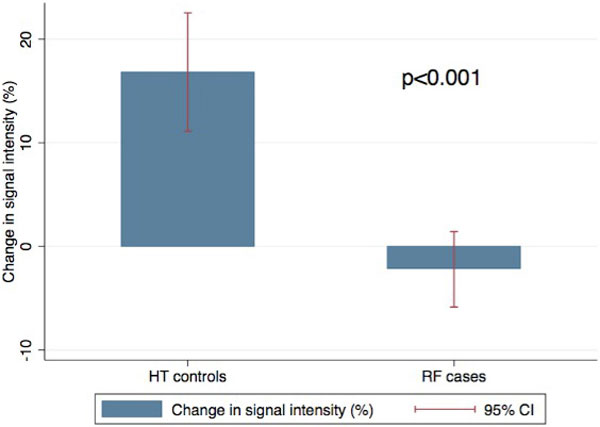
**Global Myocardial Oxygenation (BOLD SI Change)**.

Baseline clinical characteristics were similar in both CRF case and HT control groups, except higher body mass index in HT group (p = 0.02). Left and right ventricular dimensions and functions were similar. Interventricular septal thickness and LV mass were similar in both RF case and HT control groups (LV septum: 1.2 ± 0.1 cm RF vs 1.2 ± 0.1 cm HT, p > NS; LV mass index 78 ± 6 g/m2 RF vs 63 ± 4 g/m2 HT, p > NS). Rate Pressure Product (RPP) was similar in both groups. Global myocardial BOLD SI change was significantly lower in RF case group compared to HT control group (-1.79 ± 9.13 vs 17.36 ± 9.19, p = 0.0004) (Figure 1). BOLD SI Change globally and in the LAD and RCA coronary artery territory level were significantly lower in patients with renal failure (Table 1), (although did not reach statisticalsignificance in the left circumflex territory).

**Table 1:** Myocardial Oxygenation in Left Anterior Descending (LAD), Left Circumflex (LCx) and Right Coronary Artery (RCA) Territories

BOLD SI Change	RF Cases (n = 16)	HT Controls (n = 6)	p
Global Myocardium	-1.79 +/- 9.13	17.36 +/- 9.19	0.0004

LAD	-3.08 +/- 14.37	18.97 +/- 2.39	0.0016

LCx	1.49 +/- 13.66	14.29 +/- 18.74	0.097

RCA	-5.03 +/- 8.47	17.21 +/- 12.21	0.0001

## Conclusions

Chronic renal failure patients have global reductions in myocardial oxygenation even controlling for the degree of LVH. BOLD CMR is a promising tool to detect myocardial ischemia in the renal failure population, and may form a novel risk predictor of cardiovascular prognosis.

## Funding

None.

